# An Assessment of Initial Leaching Characteristics of Alkali-Borosilicate Glasses for Nuclear Waste Immobilization

**DOI:** 10.3390/ma12091462

**Published:** 2019-05-06

**Authors:** Osama M. Farid, Michael I. Ojovan, A. Massoud, R.O. Abdel Rahman

**Affiliations:** 1 Reactors Department, Nuclear Research Center, Atomic Energy Authority of Egypt, Cairo 13759, Egypt; usamafa98@hotmail.co.uk; 2 Department of Materials Science and Engineering, The University of Sheffield, Sheffield S1 3JD, UK; m.ojovan@sheffield.ac.uk; 3 Department of Radiochemistry, Lomonosov Moscow State University, 119991 Moscow, Russia; m.i.ojovan@gmail.com; 4 Chemistry Unit of Cyclotron, Nuclear Research Center, Atomic Energy Authority of Egypt, Cairo 13759, Egypt; ayman_mass@yahoo.com; 5 Hot Laboratory Center, Atomic Energy Authority of Egypt, Cairo 13759, Egypt

**Keywords:** fractional release, alkali borosilicate glass, leaching processes, modeling

## Abstract

Initial leaching characteristics of simulated nuclear waste immobilized in three alkali- borosilicate glasses (ABS-waste) were studied. The effects of matrix composition on the containment performance and degradation resistance measures were evaluated. Normalized release rates are in conformance with data reported in the literature. High Li and Mg loadings lead to the highest initial de-polymerization of sample ABS-waste (17) and contributed to its thermodynamic instability. Ca stabilizes non-bridging oxygen (NBO) and reduces the thermodynamic instability of the modified matrix. An exponential temporal change in the alteration thickness was noted for samples ABS-waste (17) and Modified Alkali-Borosilicate (MABS)-waste (20), whereas a linear temporal change was noted for sample ABS-waste (25). Leaching processes that contribute to the fractional release of all studied elements within the initial stage of glass corrosion were quantified and the main controlling leach process for each element was identified. As the waste loading increases, the contribution of the dissolution process to the overall fractional release of structural elements decreases by 43.44, 5.05, 38.07, and 52.99% for Si, B, Na, and Li respectively, and the presence of modifiers reduces this contribution for all the studied metalloids. The dissolution process plays an important role in controlling the release of Li and Cs, and this role is reduced by increasing the waste loading.

## 1. Introduction

Radioactive waste disposal is considered to be the last step (end point) in radioactive waste management systems [1,2,3]. The design of both geological and near-surface disposal facilities relies on the application of passive safety functions to ensure the containment and confinement of the radiological hazards of these wastes, where the wastes are isolated for periods sufficient to allow for radioactive decay of the short-lived radionuclides and limit the release of long-lived radionuclides [1,2,4,5]. To ensure safe performance of these facilities throughout their life cycles, assessment studies have to be conducted to support the decision-making process. In these assessments, temporal evolution of engineering barriers and the dynamic nature of hydrological and biological subsystems in the host environment are considered by applying a modular approach [3,5,6]. In this approach, the disposal system is divided into near- and far-field subsystems that are subsequently divided into their main components [3].

The waste-immobilizing matrix is the main component of the near-field subsystem. Its main safety functions are to ensure structural stability, resist degradation, and limit water ingress and radio-contaminant releases. Several waste matrices have been proposed to stabilize the radioactive/nuclear wastes, including, cement-, bitumen- and polymer-, glass-, and ceramic-based matrices [4,6,7,8,9,10,11,12,13,14]. The main safety function of glass waste matrices is to slow down radionuclide releases from a geological disposal facility [15]. In this respect, two performance indicators are used to assess the quality, reliability, and efficiency of the waste matrices, namely the glass–water reaction rate and the radionuclide leach rate that ensure the degradation resistance and containment ability of the matrices, respectively. These indicators are evaluated by conducting leaching experiments that simulate leaching conditions under conservative disposal conditions.

Generally, leaching characteristics of radioactive/nuclear waste matrices are highly dependent on the chemical compositions of the waste matrices and leaching experimental conditions [6,7,8,9,11,12,13,14,15,16,17]. A huge research effort was directed at studying the leaching characteristics of glass-based waste matrices using static and dynamic leaching experiments, i.e., PCT (product consistence test), MCC (Material Characterization Center), and single pass flow through tests, by investigating different waste matrices and leachant compositions at varying pH and temperature values and leachant-to-waste volumes [10,11,12,13,14,15,16,17,18,19,20,21,22,23,24,25]. These studies identified hydrolysis, ion exchange, diffusion, dissolution, and re-precipitation as the main corrosion processes for glass structural elements that led to glass degradation [10,11,12,13,14,15,16,17,18,19,20,21,22,23,24,25]. The overall temporal evolution of the glass waste matrix was attributed to these processes and their interactions and is conventionally divided into four [11,12,13,14] or three [16,17,24] basic stages, namely initial/forward (inter-diffusion and hydrolysis), residual/final, and resumption of alteration.

Safety assessment studies for the glass waste matrices are based on kinetic models to predict temporal variation in radio-contaminant releases and glass degradation [17,23]. Long-term assessment studies are challenged by the quantification of potential formation of zeolites and their roles in enhancing long-term glass degradation, whereas short-term assessments are challenged by the dynamic changes in the leachant chemical composition and glass surface area [11,12,13,14,16,17,23,24]. In addition, the initial leaching stage is characterized by the fastest leaching rates that result from contributions of different leaching processes [11,12,13,14,17,24,26]. An understanding of the leaching characteristics of all the matrix elements at this stage and an assessment of initiating leaching processes can help in predicting and controlling the releases at subsequent stages of the degradation process.

Borosilicate glasses (BSs) were proposed as nuclear-waste-immobilizing matrices because of their ability to incorporate a wide variety of metal oxides, high waste loading, physical and radiological stability, and simplicity of production [10,11,12,13,14,27]. Alkali modifiers can affect the durability of borosilicate matrices as a result of a boron anomaly and formation of non-bridging oxygen (NBO) [10,11,28]. Table 1 summarizes normalized release rates for different contaminants and structural elements for different alkali-borosilicate waste glass (ABS) matrices [29,30,31,32,33]. In this work, the short-term temporal evolution of glass-waste matrices will be investigated by assessing the initial glass leaching characteristics for all the matrix constituents in three borosilicate waste glasses. The aim is to identify the effects of waste loading and matrix modification on the containment performance and degradation resistance and vindicate the controlling leaching mechanism for each metal group. In this context, we investigate short-term MCC1 leaching characteristics of three borosilicate waste glass matrices that represent modified/unmodified vitreous waste forms of varying metal oxide loading. Temporal changes in the leaching solutions’ composition will be presented for all the matrices constituents, glasses composition evolution will be traced by calculating the non-bridging oxygen (NBO), and the associated degradation will be evaluated by calculating the corresponding altered glass fraction (δAGF(t)) and alteration thickness (ET). The hydration free energies of the glasses will be calculated to have insights into the effect of the chemical composition on the glass stability and identify the role of the structural elements, modifiers, and different waste constituents on the initial thermodynamic stability of the matrices. The leaching mechanisms of all the studied elements will be identified, and corresponding leaching parameters will be estimated. Finally, the contribution of each leaching process to the short-term releases will be presented and linked to the structure of the glasses. The main text is divided into two sections; the first (Section 2) presents the glass preparation, leaching test, free energy of hydration calculation, and leaching mechanism evaluation procedures and the second (Section 3) presents the results and discussions of the experimental and theoretical investigations.

## 2. Materials and Methods

### 2.1. Glasses Preparation

Alkali-borosilicate glasses were prepared using the melt quenching technique, where powders were mixed, as indicated in Table 2, Table 3 and Table 4, and milled to obtain homogeneous batches. These samples simulate the performance of ABS-17% Magnox (ABS-waste (17)), Modified ABS-20% Magnox (MABS-Waste (20)), and ABS-25% Mixed oxide (ABS-Waste (25)). The powder mixes were melted in a platinum crucible at 1060 °C for 1 h and stirred for 4 h before casting into blocks using a preheated stainless steel mould. Glasses were allowed to cool before being placed into an annealing furnace at 500 °C for 1 h then to cool to room temperature at a rate of 1 °C/min. The glasses were kindly supplied by Dr. Cassingham, N.C. and Prof. Hyatt, N.C., Immobilization Science Laboratory, The University of Sheffield, Sheffield, UK.

### 2.2. Leaching Test

Glass leaching was assessed by conducting an MCC1 (ASTM C1220-10) static leaching test [11], where glass coupons of 1 × 1 × 0.5 cm^3^ were immersed in deionized water in Perfluoroalkoxy (PFA) vessels. The test was performed at 90 °C using a constant surface area to volume ratio (S/V) 10 m^−1^ for all samples studied. The spectroscopic analyses of the leachants as a function of time were conducted using inductively coupled plasma optical emission spectroscopy (ICP-OES). The experimental data (average of triplicates) were used to calculate four performance measures that represent temporal changes in the leaching solution composition and glass waste matrices compositions, i.e., normalized release rates (NR_i_, mg·m^−2^·d^−1^) and non-bridging oxygen (NBO), and its corresponding degradation, i.e., altered glass fraction (δAGF(t)) and altered thickness (ET(t), μm) [6,10,19,22,23,33,34]:

(1) $${\mathrm{NR}}_{i}=\frac{C_{i}V}{f_{i}S\Delta t}$$

(2) $$\mathrm{NBO}=2\left(R_{2}O+\mathrm{RO}\right)+6R_{2}O_{3}-2\left({\mathrm{Al}}_{2}O_{3}+{\mathrm{Fe}}_{2}O_{3}\right)+4{\mathrm{RO}}_{2}$$

(3) $$\delta \mathrm{AGF}\left(t\right)=\left(C_{B_{t}}-C_{B_{t-1}}\right)\left(\frac{V}{m_{B}}\right)$$

(4) $$\mathrm{ET}\left(t\right)=\left(1-{\left(1-\mathrm{AGF}\left(T\right)\right)}^{\frac{1}{3}}\right)\left(\frac{3}{\rho \times \mathrm{SA}}\right)$$

where C_i_ is the measured element (i) concentration in leachant released at a specified time t (g/m^3^), V and S are the leachant volume (m^3^) and sample surface area (m^2^), respectively, f_i_ is the fraction of the element in the sample, Δt is the time change, R_x_O_y_ is the metal oxide amount, m_B_ is the mass of boron (g), ρ is the glass density (g/cm^3^), and SA is the specific surface area (m^2^/g).

### 2.3. Free Energy of Hydration

Leaching behavior can be viewed as a combination of two subsequent reactions. The first is the waste matrix hydration followed by elemental transport through the matrix and interaction with the leachant solution. Subsequently, the tendency to undergo a hydration reaction could be seen as an indication of the waste matrix instability. The hydration free energy (ΔG) for glass waste matrices was correlated to the thickness of the altered glass, pH, Eh, and former normalized release rates [29,30,35]. The free energy of hydration reaction is expressed as an additive function of individual glass units’ hydration free energies (ΔG_i_), as follows:

(5) $$\Delta G=\underset{i}{\sum }x_{i}\Delta G_{i}$$

where x_i_ is the mole fraction of an individual glass unit (i). The hydration free energy was determined based on the assumption that the glass matrix is homogenous and the presence of crystalline phases, i.e., iron spinel, is of negligible effect on the hydration. This negligible effect is attributed to their isotropic nature that minimizes grain boundary dissolution [26]. All the metal oxides were converted into silicates except silicon, boron, aluminum, and iron and the individual hydration free energy at 90 °C was obtained as indicated by Perret et al. [35].

### 2.4. Leaching Mechanisms Evaluation

Glass leaching mechanisms were evaluated based on the analysis of the experimental data to a collective model that represents the cumulative leach fraction (CLF_i_) of the structural elements, modifiers, and waste oxides as superimposed leaching processes that include a first-order reaction exchange between the leaching solution and bounded element on the matrix or the formed colloides, bulk diffusion of elements throughout the matrix, congruent dissolution, and instantaneous release of loosely bounded element from the surface [6,7,9,10,11,12,13,14,36]:

(6) $${\mathrm{CLF}}_{i}=Q_{\mathrm{Oi}}\left(1-e^{-K_{i}t}\right)+\left(\frac{S}{V}\right)\left(2\sqrt{\frac{D_{i}t}{\pi }}+U_{i}t\right)+C,$$

where Q_oi_ is the initial exchangeable fraction of element on the surface of the waste form, K_i_ is the rate constant for the exchange reaction (h^−1^), U_i_ is the glass network dissolution rate (m·h^−1^), and D_i_ is the effective diffusion coefficient of the element (m^2^·h^−1^) .This equation is used in conditions when saturation effects are not important, such as the initial stage of glass dissolution.

## 3. Results and Discussion

### 3.1. Leaching Behavior

**Figure 1:**
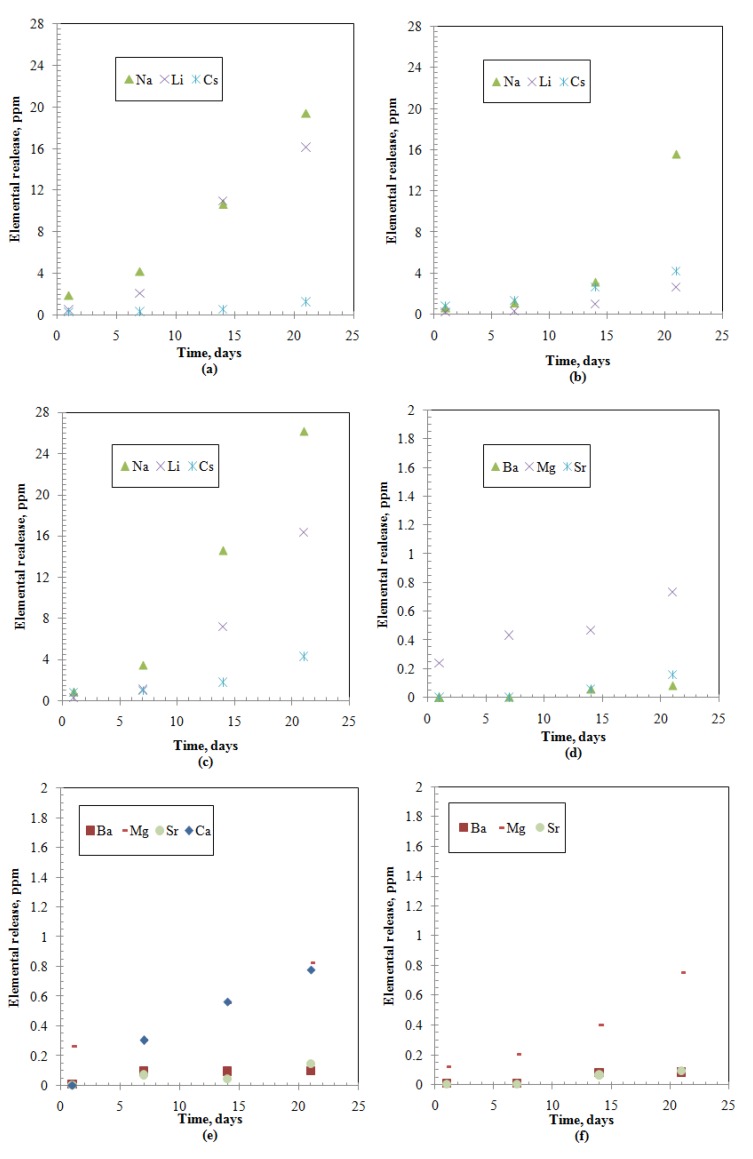
Elements releases from the studied samples: (**a**) Group I-Alkali-Borosilicate (ABS)-Waste (17); (**b**) Group I- Modified Alkali-Borosilicate (MABS)-Waste (20); (**c**) Group I-ABS-Waste (25); (**d**) Group II-ABS-Waste (17); (**e**) Group II-MABS- Waste (20); (**f**) Group II-ABS-Waste (25).

**Figure 2:**
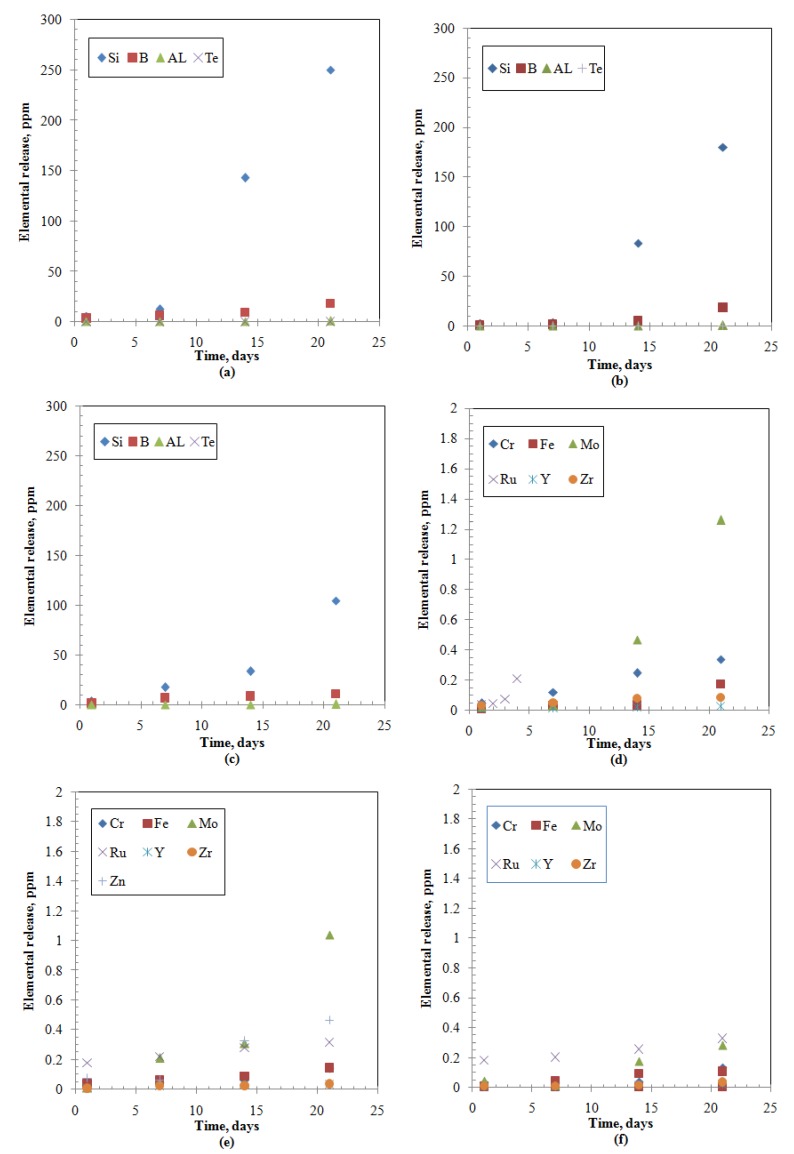
Elemental releases from the studied samples: (**a**) Metalloid and post-transition-ABS-Waste (17); (**b**) Metalloid and post-transition-MABS-Waste (20); (**c**) Metalloid and post-transition-ABS-Waste (25); (**d**) Transition-ABS-Waste (17); (**e**) Transition-MABS-Waste (20); (**f**) Transition-ABS-Waste (25).

**Figure 3:**
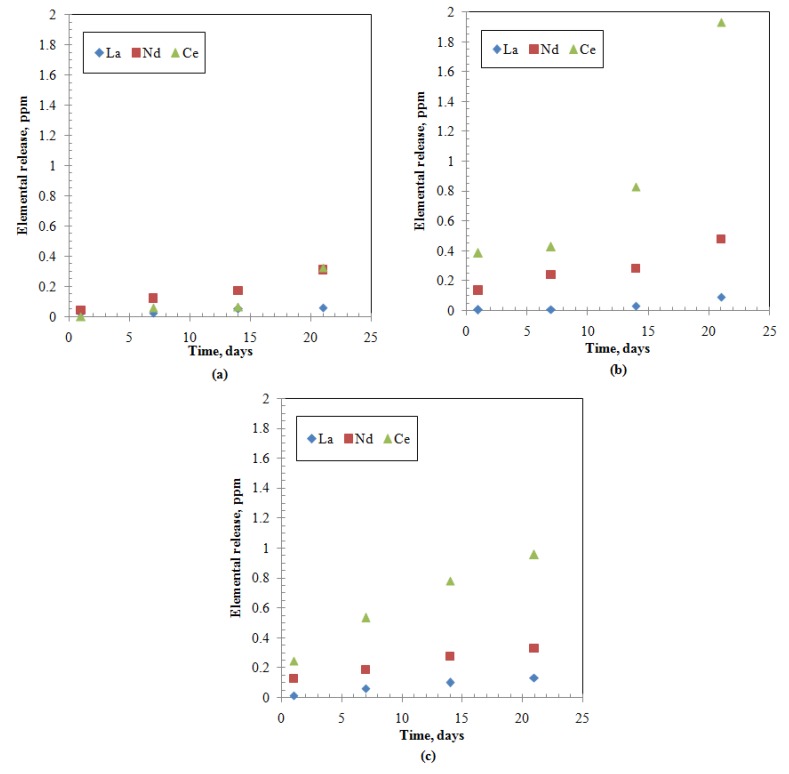
Rare earth elements releases from the studied samples: (**a**) ABS-Waste (17); (**b**) MABS-Waste (20); (**c**) ABS-Waste (25).

Elemental releases (Ci) for all the studied elements show an increasing pattern with time characterized by an initial slow portion (within 7 days) followed by steep increase rates (Figure 1, Figure 2 and Figure 3). The release of alkaline earth metals from MABS-Waste (20) and ABS-Waste (25) is characterized by very slow rates (Figure 1e,f) and their normalized release rates are in conformance with published data for different ABS-waste matrices [30]. Glass formers have higher releases than that of Al and Te (Figure 2), and increasing the metal oxide loading led to a reduction in the releases for metalloid, post-transition, and transition elements (Figure 2). Finally, for rare earth elements, releases are characterized by a slow increase as time passes (Figure 3). The normalized release rates of alkali metals (Table 5, Table 6 and Table 7) are in conformance with reported data for ABS-Reactor Bolshoy Moshchnosty Kanalny (RBMK), ABS-Water-Water Energetic Reactor (WWER), K-26, and composite glass [31,32]. Sample ABS-waste (17) has the highest normalized release rates for most of the studied elements, whereas ABS-waste (25) has the lowest normalized release rates for formers, alkaline earth elements, and transition elements. The low values of boron’s normalized leach rates suggest the formation of smectite alteration phases in the three samples at extended leaching times [37]. From the abovementioned data, it can be concluded that the releases for all studied elements are monotonically increasing with time, and the changes in the slope of the release-time represent a possible change in the controlling leaching mechanisms [7,9,11,12,13,14,27].

**Figure 4:**
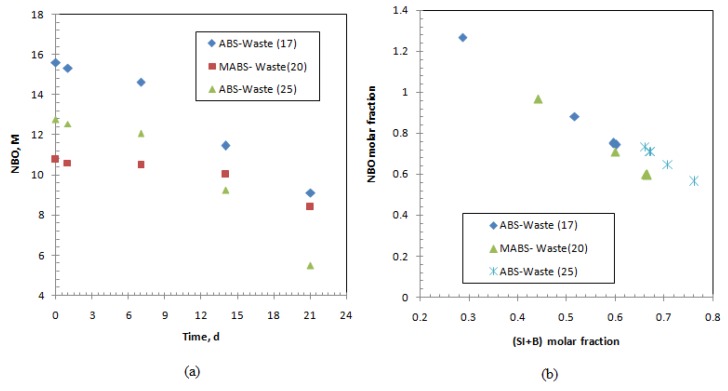
The evolution of glass matrix composition during the leaching process: (**a**) temporal changes in non-bridging oxygen (NBO); (**b**) The NBO fraction as a function of former fraction.

NBO are formed in ABS-waste matrices due to the presence of alkali modifiers and the waste metal oxides (Equation (2)); a higher value of NBO fraction is indicative of glass matrix de-polymerization [15,34]. The silicon-to-boron (Si/B) ratio for all studied samples is greater than 2, which highlights the role of NBO in glass degradation and refers to the neglected effect of cluster detachment in this process [38]. ABS-waste (17) has the highest de-polymerization potential due to the presence of the largest fraction of higher field strength elements, i.e., Li and Mg represent 16.9%, that enhances BO_3_ and NBO cluster formation [10,19,39,40]. The NBO are reduced during the progress of the leaching process due to modifiers and waste metal oxides releases; the overall NBO reduction is in the order ABS-Waste (25) > ABS-waste (17) > MABS-waste (20) (Figure 4a). It is noted that the MABS-waste (20) sample, which is the highest polymerized matrix, has a different NBO reduction pattern that is characterized by its slowest rate of NBO reduction within the first week. This behavior is accompanied by reduced silicon and boron releases (Figure 2b) and nearly unleached Zn (Figure 2e). This can be attributed to the nature of modifier incorporation in the matrix, where Ca incorporated in the vitreous structure of the matrix to compensate for the charge and Zn formed a spinel crystalline structure [10]. Although calcium has high field strength and is involved in the formation of NBO, the enhanced highest polymerization of this matrix might be related to the following [37,39,40,41]:

- The ratio between alkali and alkaline elements to boron is greater than 1, which led to enhanced calcium stabilization;
- Ca silicate has a lower hydration free energy compared to alkali elements silicates, which led to lower calcium hydration and subsequently a more stable sample.

Figure 4b quantifies the effect of glass former fraction evolution during the leaching process on glass matrix de-polymerization. A reducing linear pattern is noted, where the lowest NBO fractions (0.6–0.76) are noted for the unleached samples (higher glass former fraction content). As the leaching process continues, the glass former fraction is reduced and the NBO fraction increases. The linear dependency between the formers and NBO fractions indicates that both silicon and boron sites are linked to NBO [37]. ABS-waste (17) has the highest NBO fraction, which explains its higher normalized release rate, whereas the ABS-waste (25) has the lowest fraction. The linear regression coefficients are in the range (0.994–0.999), where the highest NBO fractions of fully degraded samples are in the range (1.7–1.8), and the degradation slope is in the order ABS-Waste (25) < MABS-waste (20) < ABS-waste (17).

Table 8 shows glass matrix degradation measures. It reveals that the fraction of the degraded glass increases with time and the highest degraded sample is ABS-waste (17), which is more stable than that of international simple glass [22]. The calculated alteration thickness for modified glass is similar to that of the experimentally deduced value of sample MABS-waste (20) [10]. The relations between the calculated ET values and the leaching time (t) and the Boron releases in terms of cumulative leach fraction of boron (CLF_B_) were calculated via regression as illustrated in Table 8. The alteration thickness increases exponentially as the leaching period for ABS-waste (17) and MABS-waste (20) samples increases, whereas a linear dependence is noted with time for the ABS-Waste (25) sample. The linear dependence between the alteration thickness and the time was noted for some glass samples during a very short leaching experiment (t < 8 h) [19]. This indicates that the mechanism that controls that leaching process within the studied period is not diffusion [42,43]. It should be noted that the formed alteration layer is inhomogeneous, as it is formed under non-equilibrium conditions, and the main driving degradation force is the matrix chemical composition within the studied period [44]. The investigations of the relation between alteration layer thickness and boron cumulative leach fraction shows a linear dependency, where the formation of the alteration layer is the most sensitive in the case of ABS-waste (17).

### 3.2. Hydration Free Energy of the Studied Matrices

**Figure 5:**
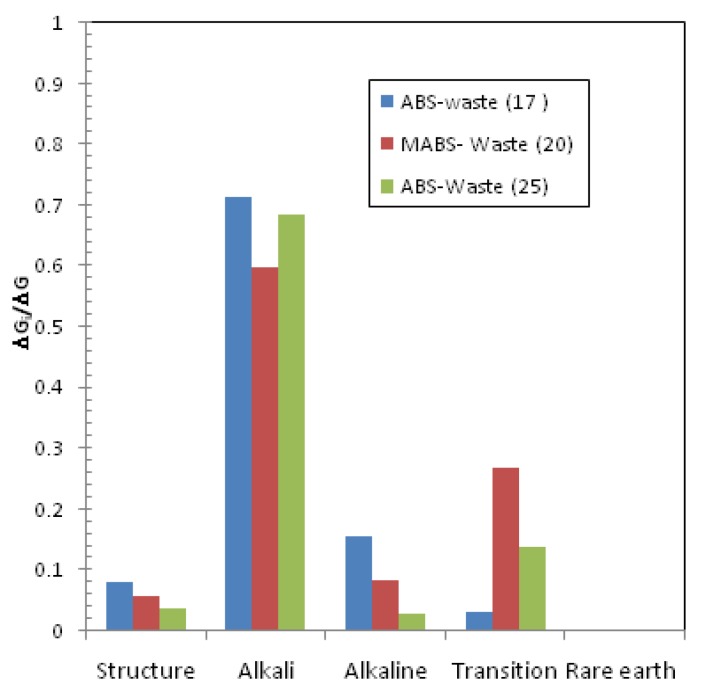
Contribution of the waste matrix constituents to the hydration free energy.

The hydration free energies of the matrices were −6.7, −5.45, and −6.0 kcal/mol for ABS-waste (17), MABS-waste (20), and ABS-Waste (25), respectively. These values refer to the spontaneous nature of the hydration reaction that is reduced with increasing the metal oxide loading. The use of calcium and zinc additives has reduced this spontaneous nature of the reaction. The contribution of the glass constituents to the hydration free energy is shown in Figure 5. It is clear that the presence of the rare earth elements does not contribute to the hydration reaction, which is attributed to their low content and small hydration energy. These elements could be used to stabilize the hydration reaction. Alkali metals have the highest contribution to the hydration reaction and this contribution is reduced by increasing the metal oxide loading and additive presence. Transition metals have considerable effect on the hydration reaction, and this effect increases as the metal oxide loading increases. The contribution of alkaline metals, metalloids, and post-transition elements to the hydration reaction is slightly affected by the metal oxide loading or the additive presence. It should be noted that the contribution of Li and Mg to the overall hydration free energy of the sample ABS-waste (17) represents 46.73%, which is reduced to 18.92% and 27.22% for the samples MABS-waste (20), and ABS-Waste (25), respectively. So, it could be concluded that the presence of Li and Mg had led to the higher degradation of the sample ABS-waste (17), as their presence increases the thermodynamic instability of the sample by increasing the hydration free energy. Reported studies indicated that the presence of Na- and Mg-silicates have reduced the glass stability [35].

### 3.3. Leaching Mechanism of Structural Elments and Metaloids

**Figure 6:**
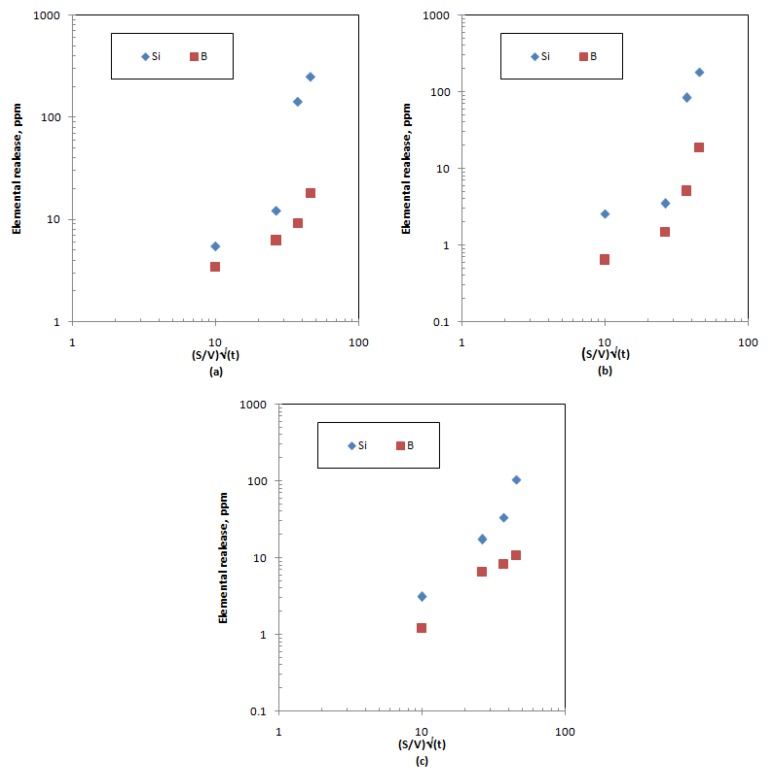
Preliminary investigation of structural element leaching mechanisms for samples: (**a**) ABS-waste (17); (**b**) MABS-waste (20); (**c**) ABS-waste (25).

The controlling leaching mechanism is preliminarily screened by plotting the release of structural glass elements (Si and B) as a function of square root time; linear plots indicate the diffusion-controlled process [7,9,11,24,26,43]. Visual examination of the experimental patterns for both silicon and boron show non-linear dependency between elemental release in the leachant and the square root of time for the sample that contains the lowest metal oxide loading. As the waste loading increases, a weak linear dependency starts to appear (Figure 6a–c). The mask of the linear dependency reflects that the dominant leaching process is congruent dissolution not diffusion [10,24,43]. This indicates that, as the metal oxide loading increases, the diffusion through the matrix or the ion-exchange mechanism plays an important role in determining the leaching characteristics. An earlier study on the characterization of sample MABS-waste (20) showed that ion-exchange contributed to the leaching mechanism after 7 days of the leaching experiment [10].

**Figure 7:**
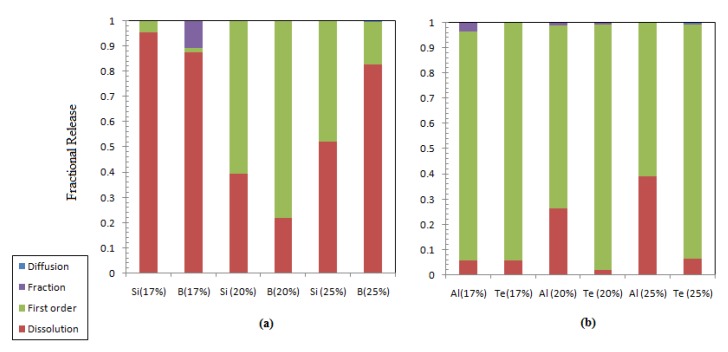
Contribution of different leaching processes to the fractional release: (**a**) structural element; (**b**) post-transition and other metalloid elements.

To identify the controlling mechanism and the effect of the metal oxide loading on the mechanisms, the experimental data were fitted to the collective leaching model. Table 9, Table 10 and Table 11 list the fitting parameters for metalloids and post-transition elements incorporated in the waste matrices; it is obvious that diffusion only contributes to the release of boron (i.e., the diffusion coefficient has a significant value) from the highest metal oxide waste. Silicon release takes place via dissolution and a first-order reaction independently on the mixed oxide incorporation percentage. This also applies to boron release, except for low metal oxide incorporation (sample ABS-waste (17%)), where some fraction of loosely bounded boron is released. The loosely bounded boron fraction is independent of time and could be related to the reduced polymerization due to the presence of Li and Mg [38]. The maximum dissolution rates for both elements are the highest for the ABS-waste (17) sample and decreased with increasing the metal oxide loading. Figure 7a shows that linear dissolution is the main leaching mechanism that causes the release of both structural elements from ABS-waste matrices (17 and 25%). This finding is in conformance with the interfacial dissolution-reprecipitation theory that proposes dissolution of structural elements as the controlling process in the initial stage of glass degradation [39,40,45]. For the MABS-waste (20) matrix, the main leaching process is a first-order reaction, which could be attributed to the absence of a large ring of silica tetrahedrons that limit the water diffusion into the matrix as a result of matrix modification [40,46]. It is clear that, as the waste loading increases, the contribution of the dissolution process to the overall release of silicon and boron decreases by 43.44 and 5.05%, respectively, and the presence of modifiers reduces this contribution by 56.19 and 65.60% for silicon and boron, respectively.

On the other hand, the fractional releases of Al as a post-transition element and Te as a metalloid waste component are mainly controlled by the first-order reaction (Figure 7b). A small fraction of Al release could be attributed to the instantaneous leaching of loosely bound Al in the sample (ABS-Waste (17)). This fraction was not noted for the other samples; this might be due to the effect of the modifier and the decreased Al loading, where a higher Al loading can create an Al cluster and large silicon rings [38]. The contribution of the dissolution process to Te release is fairly constant independently of its loading, except for the modified sample that has a lower contribution to the dissolution.

### 3.4. Leaching Characteristics of Alkali and Alkaline Earth Metals

**Figure 8:**
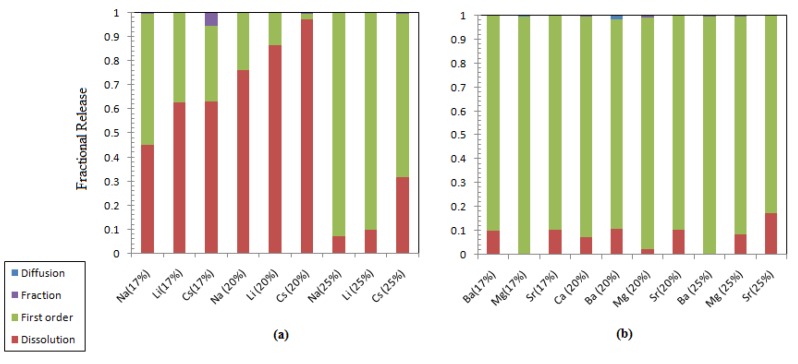
Contribution of different leaching processes to the fractional release: (**a**) alkali elements; (**b**) alkaline earth metals.

Table 12, Table 13 and Table 14 list the estimated leaching parameters, revealing that the diffusion of alkali and alkaline earth metals does not play any role in controlling their leaching behavior at any waste loading. To quantify the role of each mechanism in the overall cumulative leaching fraction, the contribution of each mechanism was plotted and is shown in Figure 8. It is clear that congruent dissolution of Li and Cs is the major mechanism for ABS-waste (17) and MABS-waste (20). As the metal oxide loading increases, the first-order exchange reaction becomes a dominant leaching process. The increase in the waste loading from 17 to 25% reduced the contribution of the dissolution mechanism to the release by 38.07, 52.99, and 31.25% for Na, Li, and Cs, respectively. Alkaline metal leaching is controlled by a first-order exchange reaction. This notable change in the controlling leaching process for alkali and alkaline metals could be attributed to the higher field strength of the alkaline metals that leads to glass stabilization [46].

### 3.5. Leaching Characteristics of the Transition and Rare Earth Elements

**Figure 9:**
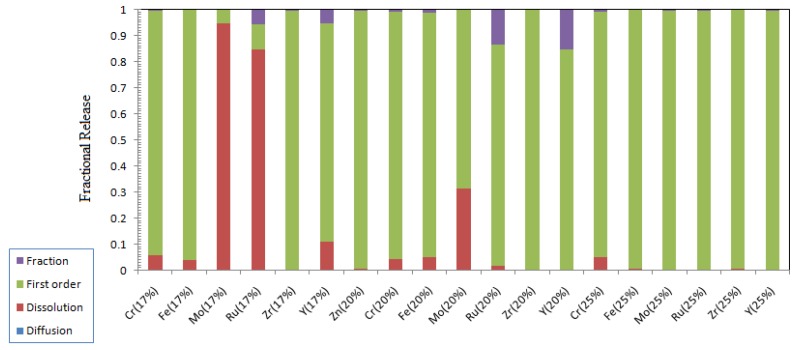
Contribution of different leaching processes to the fractional release of transition metals.

**Figure 10:**
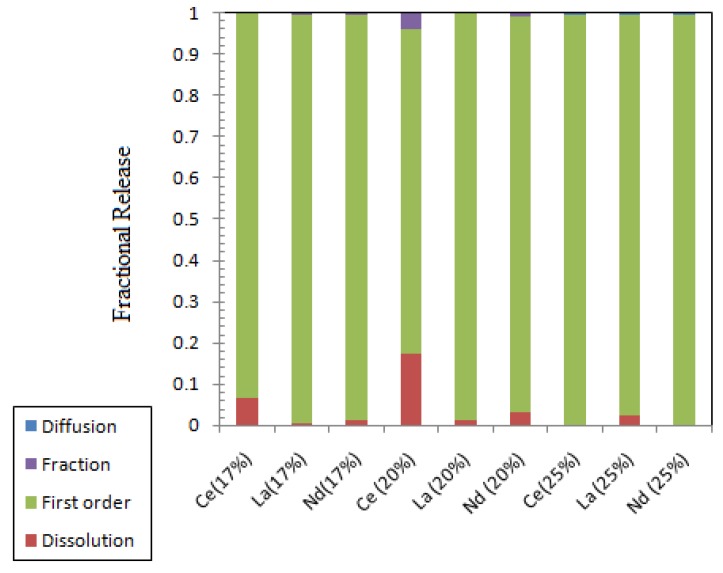
Contribution of different leaching processes to the fractional release of rare earth elements.

The leaching parameters as estimated from the nonlinear regression of the experimental data to the collective model for transition and rare earth elements are given in Table 15, Table 16 and Table 17, and the contribution of each leaching process to the overall release fraction is presented in Figure 9 and Figure 10. Ru and Mo release from ABS-Waste (17) sample is only controlled by the dissolution, and the rest of the releases are controlled by the first-order model. Increasing the metal oxide loading can lead to the formation of spinels that are used to immobilize transition metal ions [10].

## 4. Conclusions

Leaching characteristics of different structural elements, modifiers, and waste components were investigated for three alkali-borosilicate**-**mixed oxide glasses that represent different waste loadings. The main concluding remarks from this work are as follows:

1. The normalized release rates of the studied elements are in conformance with data reported in the literature for borosilicate waste glass matrices.
2. Elemental releases monotonically increase with time; the changes in the slope of the Release-Time represent a possible change in the controlling leaching mechanism.
3. The high incorporation of Li and Mg in the ABS-waste (17) glass led to a high de-polymerization of glass and contributed to the thermodynamic instability of the matrix.
4. The MABS-waste (20) glass has the slowest rate of NBO reduction due to the incorporation of Ca as matrix modifier of low hydration free energy which increased the thermodynamic stability against a hydration reaction.
5. Rare earth elements could be used to stabilize the glass hydration reactions.
6. The alteration thickness increases exponentially with increasing the leaching period for the ABS-waste (17) and MABS-waste (20) samples, whereas a linear dependence is noted with time for the ABS-Waste (25) sample.
7. The alteration layer thickness is linearly dependent on boron’s cumulative leach fraction and the formation of the alteration layer is the most sensitive in the case of ABS-waste (17) glass.
8. As the waste loading increases, the contribution of the dissolution process to the overall fractional release of structural elements decreases and the presence of modifiers reduces this contribution for all the studied metalloids.
9. The use of Zn and Ca modifiers could reduce the instantaneous release of Al.
10. The initial fractional release of alkaline earth metals and transition and rare earth elements is mostly controlled by the first-order reaction process, with notable exceptions for Mo and Ru.

## Figures and Tables

**Table 1:** The normalized release rate (mg·m^−2^·d^−1^) of different elements from different alkali-borosilicate glasses matrices (ABS), including calcined Prototype Fast Reactor-Raffinate (PFR), Reactor Bolshoy Moshchnosty Kanalny-concentrate (RBMK), Water-Water Energetic Reactor-concentrate (WWER), RBMK-evaporator concentrate (K-26), High Level Waste Simulant (BS-5), and PyroGreen salt waste (PG).

ABS Glass Waste		PFR	RBMK	WWER	K-26	BS-5	PG
Test Type		PCT	ISO-6961	PCT	PCT	PCT	Field Data
Alkali	Na	16.9–21.7	10^1^–10^2^	10^2^	59.3–90.9	378	1.42–8.57
	Li	-	-	-	-	-	5.7–37.14
	Cs	-	10^1^–10^2^	10^2^	-	-	-
Alkaline earth metal	Ca	3.62–5.89	-	-	-	-	-
	Sr	-	10^0^–10^1^	10^1^	-	-	-
Post-transition	Al	0.29	-	-	-	-	-
Transition	Mo	4.44–6.38	10^0^–10^−1^	10^0^	-	-	-
	Ba	1.47–4.43			-	-	-
	Cr	0.16–0.35			-	-	-
Metalloid	Si	7.18–8.4	-	-	28.1–29.3	174	4.28–17.1
	B	32.4–33.3	<10^−1^	<10^−1^	31.3–40.5	435	1.42–18.57
Rare earth elements		-	10^−1^	10^−1^	-	7.11	-
Reference		[29]	[30]	[30]	[31]	[32]	[33]

**Table 2:** Chemical composition of the studied glasses (structural elements and modifiers).

Compound	SiO_2_	B_2_O_3_	Na_2_O	Li_2_O	CaO	ZnO	Total
ABS-Waste (17)	50.200	15.400	8.800	8.700	--	--	83.100
MABS-Waste (20)	44.260	17.950	9.010	2.110	1.390	4.430	79.150
ABS-Waste (25)	46.280	16.430	8.330	3.980	--	--	75.020

**Table 3:** Chemical composition of the studied glasses (waste components: alkali, alkaline, post-transitions, and metalloids).

Compound	Alkali	Alkaline Earth Metals				Post-Transitions and Metalloids		
	Cs_2_O	BaO	MgO	SrO	Total	Al_2_O_3_	TeO_2_	Total
ABS-Waste (17)	0.300	0.200	8.200	0.200	8.60	3.100	0.100	3.200
MABS-Waste (20)	0.890	0.40	4.100	0.240	4.740	4.110	0.150	4.260
ABS-Waste (25)	1.590	0.470	1.610	0.410	2.490	1.910	0.280	2.190

**Table 4:** Chemical composition of the studied glasses (waste components: transitions and rare earth elements).

Compound	Transition Metals*							Rare Earth *			
	Cr_2_O_3_	Fe_2_O_3_	MoO_3_	RuO_2_	ZrO_2_	Y_2_O_3_	Total	CeO_2_	La_2_O_3_	Nd_2_O_3_	Total
ABS-Waste (17)	0.300	1.300	0.700	0.200	0.800	0.100	3.400	0.500	0.100	0.400	1.000
MABS-Waste (20)	0.630	2.790	1.320	0.520	1.240	0.160	6.660	0.960	0.520	1.530	3.010
ABS-Waste (25)	0.510	2.060	2.490	0.550	2.820	0.310	8.740	1.450	0.730	2.170	4.350

* Ni, Pr, and Gd oxides were neglected in this study.

**Table 5:** The normalized release rate (mg·m^−2^·d^−1^) for structural elements and modifiers.

Compound	SiO_2_	B_2_O_3_	Na_2_O	Li_2_O	CaO	ZnO
ABS-Waste (17)	50.814	36.024	28.301	37.940	-	-
MABS-Waste (20)	41.361	31.541	22.256	25.977	1.041	0.397
ABS-Waste (25)	22.918	20.121	40.363	32.122	-	-

**Table 6:** Normalized release rate (mg·m^−2^·d^−1^) for waste components: Alkali, alkaline, post-transitions, and metalloids.

Compound	Alkali	Alkaline Earth Metals			Post-Transitions and Metalloids	
	Cs_2_O	BaO	MgO	SrO	Al_2_O_3_	TeO_2_
ABS-Waste (17)	40.927	2.588	0.706	4.479	3.443	4.568
MABS-Waste (20)	47.539	1.288	1.587	3.447	2.038	1.493
ABS-Waste (25)	27.198	0.928	3.686	1.266	3.464	0.857

**Table 7:** Normalized release rate (mg·m^−2^·d^−1^) for waste components: transitions and rare earth elements.

Compound	Transition Metals						Rare Earth		
	Cr_2_O_3_	Fe_2_O_3_	MoO_3_	RuO_2_	ZrO_2_	Y_2_O_3_	CeO_2_	La_2_O_3_	Nd_2_O_3_
ABS-Waste (17)	15.648	1.766	12.841	6.458	0.670	2.818	3.781	6.639	8.578
MABS-Waste (20)	3.146	0.708	5.624	3.790	0.187	2.668	8.044	1.924	3.457
ABS-Waste (25)	3.590	0.696	0.811	3.726	0.071	0.702	3.852	2.017	1.670

**Table 8:** The evolution and dependency of glass degradation measures.

Glass Sample	AGF% × 10^−4^				ET (μm)			
	1 d	7 d	14 d	21 d	Time Dependency	R^2^	Boron Release Dependency	R^2^
ABS-Waste (17)	5.419	10.437	16.423	50.337	$ET=0.466e^{0.106t}$	0.969	ET = 9.032CLF_B_ − 0.842	0.976
MABS-Waste (20)	0.780	1.832	6.889	42.672	$ET=0.052e^{0.199t}$	0.983	ET = 8.023CLF_B_ − 0.235	0.989
ABS-Waste (25)	1.629	10.448	13.540	18.685	ET=0.080t+0.244	0.943	ET = 5.395CLF_B_ − 0.072	0.997

**Table 9:** Nonlinear curve fitting parameters of the cumulative leach fraction of metalloid and post-transition elements: ABS-Waste (17).

Element	D (m^2^·h^−1^), × 10^−13^	U (m·h^−1^) × 10^−7^	Q_o_,	K (h^−1^) × 10^−8^	C × 10^−4^	R^2^
Si	0	1.583	0.004	938.143	0	0.912
B	0	1.013	0.001	619.759	62.200	0.940
Te	0	0.127	0.105	49.556	0	0.830
Al	0	0.074	0.058	0.845	22.400	0.879

**Table 10:** Nonlinear curve fitting parameters of the cumulative leach fraction of metalloid and post-transition elements: MABS-Waste (20).

Element	D (m^2^·h^−1^), × 10^−13^	U (m·h^−1^) × 10^−7^	Q_o_,	K (h^−1^) × 10^−8^	C × 10^−4^	R^2^
Si	0	1.211	0.094	10.397	0	0.934
B	0	0.851	0.152	4.011	0	0.899
Te	0	0.033	0.084	0.122	7.264	0.806
Al	0	0.065	0.009	14.107	1.576	0.917

**Table 11:** Nonlinear curve fitting parameters of the cumulative leach fraction of metalloid and post-transition elements: ABS-Waste (25).

Element	D (m^2^·h^−1^), × 10^−13^	U (m·h^−1^) × 10^−7^	Q_o_,	K (h^−1^) × 10^−8^	C × 10^−4^	R^2^
Si	0	0.648	0.003	1.630	0	0.868
B	0.678	0.303	3.20 × 10^−4^	0.149	0	0.967
Te	N*	0.028	0.002	1.592	1.879	0.902
Al	0	0.102	8.05 × 10^−4^	0.747	0	0.886

N* neglected value.

**Table 12:** Nonlinear curve fitting parameters of the cumulative leach fraction of alkali and alkaline earth metals: ABS-Waste (17).

Group	Element	D (m^2^·h^−1^), × 10^−13^	U (m·h^−1^), × 10^−7^	Q_o_,	K (h^−1^), × 10^−8^	C × 10^−4^	R^2^
Alkali metals	Na	0	0.904	0.055	3.444	3.192	0.966
	Li	0	1.266	0.038	0.0001	0	0.952
	Cs	0	1.119	0.028	1.935	49.301	0.870
Alkaline earth metals	Ba	0	0.075	0.034	0.112	0	0.907
	Mg	1.570	0.009	0.223	2.407	0.975	0.914
	Sr	0	0.126	0.054	0.124	0	0.838

**Table 13:** Nonlinear curve fitting parameters of the cumulative leach fraction of alkali and alkaline earth metals: MABS-Waste (20).

Group	Element	D (m^2^·h^−1^), × 10^−13^	U (m·h^−1^), × 10^−7^	Q_o_,	K (h^−1^), × 10^−8^	C × 10^−4^	R^2^
Alkali metals	Na	0	0.576	0.009	0.287	0	0.867
	Li	0	0.752	0.006	0.558	0	0.876
	Cs	0	1.399	0.002	0.193	0.009	0.974
Alkaline earth metals	Ca	0	0.133	0.088	39.593	0.039	0.989
	Ba	9.159	0.002	8.2*10^-4^	0.196	0	0.805
	Mg	0	0.041	0.084	0.289	5.844	0.968
	Sr	0	0.093	0.041	5.769	0	0.902

**Table 14:** Nonlinear curve fitting parameters of the cumulative leach fraction of alkali and alkaline earth metals: ABS-Waste (25).

Group	Element	D (m^2^·h^−1^), × 10^−13^	U (m·h^−1^), × 10^−7^	Q_o_,	K (h^−1^), × 10^−8^	C × 10^−4^	R^2^
Alkali metals	Na	0	1.273	0.084	2.157	0	0.949
	Li	0	1.595	0.075	37.659	0	0.890
	Cs	0	0.743	0.008	3.074	0.003	0.867
Alkaline earth metals	Ba	0	0.029	0.758	0.629	0.333	0.869
	Mg	0	0.109	0.006	0.139	4.169	0.952
	Sr	0	0.042	0.001	359.254	0	0.907

**Table 15:** Nonlinear curve fitting parameters of the cumulative leach fraction of transition and rare earth elements: ABS-Waste (17).

Group	Element	D (m^2^·h^−1^), × 10^−13^	U (m·h^−1^), × 10^−7^	Q_o_,	K (h^−1^), × 10^−8^	C × 10^−4^	R^2^
Transition elements	Cr	0	0.516	0.415	2.883	16.600	0.994
	Fe	0	0.005	0.006	0.239	0	0.876
	Mo	0	0.359	0.001	1.302	0	0.917
	Ru	0	0.172	0.001	0.747	5.654	0.898
	Zr	2.047	N*	0.022	0.609	0.600	0.961
	Y	0	0.063	0.0247	0.913	15.000	0.892
Rare earth Elements	Ce	0	0.100	0.070	0.583	0	0.885
	La	0	0.027	0.241	0.003	2.326	0.926
	Nd	0	0.026	0.110	0.352	1.033	0.971

N* neglected value.

**Table 16:** Nonlinear curve fitting parameters of the cumulative leach fraction of transition and rare earth elements: MABS-Waste (20).

Group	Element	D (m^2^·h^−1^), × 10^−13^	U (m·h^−1^), × 10^−7^	Q_o_,	K (h^−1^), × 10^−8^	C × 10^−4^	R^2^
Transition elements	Zn	0	0.003	0.033	0.341	0.103	0.968
	Cr	0	0.078	0.087	2.343	8.234	0.857
	Fe	0	0.018	0.017	0.105	2.321	0.914
	Mo	1.294	0.060	0.007	0.157	0	0.962
	Ru	27.347	0.005	0.014	42.141	22.100	0.837
	Zr	0.118	N*	0.034	0.137	0	0.946
	Y	0	0	0.005	358 × 10^3^	8.762	0.953
Rare earth Elements	Ce	0	0.212	0.048	0.005	24.600	0.929
	La	0	0.005	0.020	350.372	0	0.83
	Nd	0	0.089	0.144	7.292	11.900	0.936

N* neglected value.

**Table 17:** Nonlinear curve fitting parameters of the cumulative leach fraction of transition and rare earth elements: ABS-Waste (25).

Group	Element	D (m^2^·h^−1^), × 10^−13^	U (m·h^−1^), × 10^−7^	Q_o_,	K (h^−1^), × 10^−8^	C × 10^−4^	R^2^
Transition elements	Cr	0	0.085	0.008	742.683	6.376	0.721
	Fe	N*	0.019	0.021	21.050	0	0.974
	Mo	0	0.025	0.072	9.901	0.313	0.871
	Ru	0	0.071	0.583	0.135	28.400	0.823
	Zr	0	0.023	0.018	2.098	0	0.919
	Y	0	0.002	0.015	4.655	0.128	0.815
Rare earth Elements	Ce	64.407	0.003	0.062	393.147	0.207	0.983
	La	2.602	0.045	0.010	0.135	0	0.993
	Nd	3.063	0.019	0.038	0.279	5.912	0.944

N* neglected value.
